# Clinicopathological features, treatment outcomes, and prognostic factors of angiosarcoma: a 21-year experience at one center

**DOI:** 10.1186/s13023-025-03819-9

**Published:** 2025-06-11

**Authors:** Chenyan Fang, Xiaoting Zeng, Qing Ji, Tao Zhu, Meiyu Fang, Jun Cao

**Affiliations:** 1 Department of Gynecologic Oncology, Zhejiang Cancer Hospital, Hangzhou Institute of Medicine (HIM), Chinese Academy of Sciences, Hangzhou, 310022 Zhejiang China; 2 Department of Gynecology, Charité-Universitätsmedizin Berlin, Campus Virchow Klinikum, 10117 Berlin, Germany; 3 Department of Head and Neck and Rare Oncology, Zhejiang Cancer Hospital, Hangzhou Institute of Medicine (HIM), Chinese Academy of Sciences, Hangzhou, 310022 Zhejiang China; 4 Zhejiang Chinese Medical University, Hangzhou, 310053 Zhejiang China

**Keywords:** Angiosarcoma, Treatment, Prognostic factor, Survival

## Abstract

**Background:**

Angiosarcoma is a rare, aggressive soft tissue sarcoma characterized by poor prognosis and limited treatment consensus. Our aim was to clarify the clinicopathological features, treatment outcomes, and prognostic factors of these patients to inform improved management and follow-up strategies.

**Methods:**

We retrospectively analyzed the clinical, pathological, treatment, and survival data of patients with angiosarcoma treated from 2003 to 2024 at Zhejiang Cancer Hospital.

**Results:**

A total of 128 angiosarcoma patients were included, categorized as cutaneous (*n* = 49), visceral (*n* = 35), deep soft tissue (*n* = 31), breast (*n* = 10), and bone (*n* = 3). The median progression-free survival (PFS) and overall survival (OS) were 7 and 20 months, respectively, with 2-year PFS of 21.4% and 5-year OS of 29.6%. Superficial cases had better outcomes compared to deep-seated cases, larger tumors (> 5 cm) and distant metastases predicted worse prognosis. Primary surgery improved survival in localized disease, with adjuvant radiotherapy enhancing local control but not OS. In metastatic cases, first-line systemic therapies showed that paclitaxel-based regimens were less effective than doxorubicin-based chemotherapy, and the addition of anti-angiogenic therapy did not improve outcomes.

**Conclusion:**

This study underscores the heterogeneity of angiosarcoma across different sites, emphasizing the role of surgery for localized AS, and chemotherapy remains the mainstay for advanced cases. Targeted therapies and immunotherapies offer novel promise, especially the combination strategies.

**Supplementary Information:**

The online version contains supplementary material available at 10.1186/s13023-025-03819-9.

## Background

Angiosarcoma (AS) is a rare type of sarcoma that shows vascular differentiation, with an estimated incidence of 3 cases per million annually, comprising 2–5% of all soft tissue sarcomas (STS) [1, 2].

AS can develop in any part of the body, including the skin, soft tissues, breasts, visceral organs, and bones. The skin, particularly the scalp, is the most common site [3, 4]. A 2020 U.S. survey revealed 72.3% of AS cases involved the skin, subcutaneous or breast, while visceral AS accounted for 24.4% [2]. AS can occur at any age, with cutaneous AS being more common in elderly white men. Over 90% of breast AS occur in women, primarily as secondary AS linked to radiotherapy for breast cancer [5, 6]. Radiation can induce gene mutations, complicated with chronic lymphedema, increasing AS risk. AS caused by long-term chronic lymphedema (Stewart-Treves syndrome) usually has a latency of 5–15 years after surgery and radiotherapy, warranting extended follow-up for timely diagnosis of AS [7, 8]. Other risk factors include environmental carcinogens (e.g., vinyl chloride, arsenic) and genetic familial syndromes (e.g., retinoblastoma, Recklinghausen neurofibromatosis, Maffucci disease) [9].

Early diagnosis of AS remains challenging due to the nonspecific and often innocuous clinical manifestations [10]. Current diagnostic methods include imaging, pathology, and immunohistochemistry (IHC), with molecular markers such as CD31, CD34, FVIIIR, VEGF, vWF, UEA-1 [11, 12]. Advances in gene sequencing have revealed key genetic mutations in AS, including *KRAS*, *NRAS*, *HRAS*, *PIK3CA*, *FTL4*, *TP53*, and *CDKN2A* [13]. Additionally, genes regulating angiogenesis, such as *MYC* and *FLT4* amplifications and *PTPRB* and *PLCG1* somatic mutations, play a crucial role in secondary AS [14, 15]. Notably, *MYC* amplification has been identified as a key driver in radiation induced AS, is emerging as a potential therapeutic target. Genomic studies are providing new insights for diagnosis and targeted therapies.

Due to the small number of AS patients, data from randomized trials and prospective studies are scarce, clinical experience is also limited, making it difficult to develop uniform treatment standards. Currently, for localized lesions, radical surgical resection is the most common treatment. However, there is still debate over whether R0 resection improves survival [16]. Several studies suggest that postoperative adjuvant radiotherapy improves local control and survival, but the benefits of adjuvant chemotherapy are limited [17, 18]. For unresectable or metastatic cases, chemotherapy with taxanes, anthracycline-based regimens (e.g., doxorubicin) can be considered [19]. Nevertheless, the optimal sequence of chemotherapeutic agents remains controversial.

Additionally, AS originates from vascular endothelial cells and relies heavily on angiogenesis, making angiogenesis inhibition an important therapeutic strategy. Along with the anti-VEGF monoclonal antibody bevacizumab, tyrosine kinase inhibitors (TKIs) targeting VEGFR, PDGFR and KIT pathways, such as sorafenib and pazopanib, have been gradually applied since the late 2000s [20–22]. Immune checkpoint inhibitors, including PD-1 inhibitors (pembrolizumab, nivolumab) and CTLA-4 inhibitors, have also shown therapeutic benefits [23, 24].

The prognosis for AS patients is generally poor, with a 5-year overall survival (OS) rate of 18–54% [10], and most studies reported between 30 and 40%. Key factors affecting prognosis include primary AS, age over 60, tumors located in deep tissues or viscera, lack of radical surgery, R1 resection, high tumor grade, tumors > 5 cm, and absence of multimodal therapy [10, 16, 22, 25–28].

Although AS is rare, its incidence has increased over the past 20 years [2]. The challenge of early diagnosis, high invasiveness, high rates of local recurrence and distant metastasis, poor prognosis, as well as lack of unified treatment consensus, highlight the need of more data on prognostic factors and treatment efficacy for AS patients. We conducted a retrospective analysis of the clinicopathological characteristics, treatment, and prognosis data of 128 AS patients over the past 21 years, hoping to increase the clinical sample size, identify prognostic factors, and provide guidance for clinical management of AS patients.

## Methods

### Patients

This retrospective study analyzed 128 AS patients treated at our institution between 2003 and 2024, all with available follow-up data. Diagnoses were histologically confirmed and reviewed according to 2020 World Health Organization guideline (WHO) Classification of Soft Tissue and Bone Tumours (5th ed., vol. 3), including various tumor locations (cutaneous, viscera, deep soft tissue, breast, and bone). Lesions limited to the dermis were categorized as cutaneous AS and those extending into the subcutaneous tissue were classified as deep soft tissue AS. Radiation-induced AS was diagnosed based on a history of prior radiation in the affected area or in adjacent regions. Patient characteristics, molecular profiles, treatment, and outcomes were collected from the medical records and telephone interviews. Tumor grading was performed according to the grading system of the French Federation of Cancer Centers Sarcoma Group (FNCLCC) based on tumor cell differentiation, mitotic activity, and extent of necrosis, as outlined in the American Joint Committee on Cancer (AJCC) Cancer Staging Manual (8th ed., 2017) [29].

### Immunohistochemical (IHC) studies

All cases underwent a minimal IHC panel to confirm vascular endothelial differentiation, including CD31, CD34, and F8. Nuclear markers ERG and FLI-1 were also used to support endothelial lineage in recent years. Vimentin and Ki-67 were occasionally used to assess mesenchymal origin and proliferation. To exclude mimics such as carcinoma, melanoma, and other soft tissue tumors (e.g., leiomyosarcoma, synovial sarcoma, histiocytic sarcoma), additional markers including D2-40, VEGFR, S-100, cytokeratin, EMA, CD68, CD163, SMA, desmin, h-caldesmon, and Bcl-2 were employed.

PD-L1 testing was performed in 4 patients using antibody clone 22C3 (Dako/Agilent) at a dilution of 1:50, following the manufacturer’s protocol. Combined positive score (CPS) was used, calculated as PD-L1–expressing cells (tumor cells, lymphocytes, macrophages) divided by total viable tumor cells × 100 [30]**.**

### Molecular testing

NGS was conducted in 7 patients using a 1021-gene panel (312 with full exonic, 38 with partial intronic, and 709 with partial exonic regions coverage) via FFPE and blood samples on the Gene + Seq-2000 platform. TMB was assessed in 4 cases based on somatic mutation data across the 1021-gene panel, ≥ 9 mut/Mb was defined as TMB-High (TMB-H), < 9 mut/Mb as TMB-Low (TMB-L) [31]. Microsatellite status was assessed calculated in 5 cases by targeted NGS, analyzing insertion or deletion events in microsatellite loci covered by the panel. Microsatellite stable (MSS) indicated no significant length variation, microsatellite instability-high (MSI-H) is typically defined as instability in ≥ 30% of loci or ≥ 2 of 5 markers [32].

### Treatment and outcome analysis

Efficacy of treatment was assessed per Response Evaluation Criteria in Solid Tumors (RECIST 1.1), classifying responses as complete response (CR), partial response (PR), stable disease (SD), or progressive disease (PD). Objective response rate (ORR) was defined as CR + PR, disease control rate (DCR) as CR + PR + SD.

Primary endpoints were progression-free survival (PFS) and OS, measured from treatment initiation to progression, death or last visit. PFS1, PFS2, and PFS3 referred to progression-free intervals following first-, second-, and third-line therapies. OS from the onset of metastasis was also assessed.

### Statistical analysis

Statistical analyses were conducted using statistical package for social sciences (SPSS, version 22.0). Categorical variables were analyzed with chi-square or Fisher’s exact test. Prognostic factors for PFS and OS were evaluated via univariate and multivariate Cox regression models, with results presented as hazard ratios (HRs). Survival curves were estimated using Kaplan–Meier methods and compared by log-rank test. A two-sided *P* value of < 0.05 was considered significant.

## Results

### Patient characteristics

We retrospectively analyzed 128 patients diagnosed with AS, distributed across various anatomical sites, the patient characteristics are listed in Table 1.

**Table 1:** Clinicopathologic characteristics of AS from different anatomical sites

	All patients (N%)	Cutaneous (N%)	Deep soft tissue (N%)	Viscera (N%)	Breast (N%)	Bone (N%)
Total	128 (100)	49 (38.3)	31 (24.2)	35 (27.3)	10 (7.8)	3 (2.3)
*All patients at initial diagnosis*						
Age, years (mean, range)	58.5 ± 16.2 (11–89)	66.3 ± 14.9 (26–89)	58.7 ± 13.3 (21–79)	52.2 ± 14.7 (22–77)	45.1 ± 10.8 (28–62)	45.7 ± 33.1 (11–77)
Gender						
Male	75 (58.6)	34 (69.4)	21 (67.7)	17 (48.6)	NA	3 (100)
Female	53 (41.4)	15 (30.6)	10 (32.3)	18 (51.4)	10 (100)	0 (0)
Radiation-induced						
No	121 (94.5)	49 (100)	27 (87.1)	33 (94.3)	9 (90)	3 (100)
Yes	7 (5.5)	0 (0)	4 (12.9)	2 (5.7)	1 (10)	0 (0)
Tumor grade						
Low grade	18 (14.1)	7 (14.3)	2 (6.5)	4 (11.4)	4 (40)	1 (33.3)
High grade	12 (9.4)	5 (10.2)	1 (3.2)	5 (14.3)	0 (0)	1 (33.3)
Unknown	98 (76.6)	37 (75.5)	28 (90.3)	26 (74.3)	6 (60)	1 (33.3)
Tumor necrosis						
No	34 (26.6)	15 (30.6)	7 (22.6)	8 (22.9)	3 (30)	1 (33.3)
Yes	27 (21.1)	6 (12.2)	10 (32.3)	10 (28.6)	1 (10)	0 (0)
Unknown	67 (52.3)	28 (57.1)	14 (45.2)	17 (48.6)	6 (60)	2 (66.7)
Maximum diameter of tumor, cm (mean, range)	5.4 ± 4.1 (0.2–20)	4.1 ± 2.9 (0.2–10)	5.4 ± 4.3 (0.7–20)	7.7 ± 4.9 (1–20)	3.8 ± 2.5 (1.5–9)	5.2 ± 4 (2.3–8)
Maximum diameter of tumor, cm						
≤ 5	68 (53.1)	32 (65.3)	16 (51.6)	12 (34.3)	7 (70)	1 (33.3)
> 5	43 (33.6)	13 (26.5)	8 (25.8)	19 (54.3)	2 (20)	1 (33.3)
Unknown	17 (13.3)	4 (8.2)	7 (22.6)	4 (11.4)	1 (10)	1 (33.3)
Regional lymph node metastasis						
No	109 (85.2)	43 (87.8)	27 (87.1)	27 (77.1)	9 (90)	3 (100)
Yes	13 (10.2)	4 (8.2)	4 (12.9)	5 (14.3)	0 (0)	0 (0)
Unknown	6 (4.7)	2 (4.1)	0 (0)	3 (8.6)	1 (10)	0 (0)
Distant metastasis^a^						
No	88 (68.8)	44 (89.8)	16 (51.6)	16 (45.7)	10 (100)	2 (66.7)
Yes	40 (31.3)	5 (10.2)	15 (48.4)	19 (54.3)	0 (0)	1 (33.3)
Clinical stage						
Localized	79 (61.7)	42 (85.7)	11 (35.5)	14 (40)	10 (100)	2 (66.7)
Regional^b^	9 (7)	2 (4.1)	5 (16.1)	2 (5.7)	0 (0)	0 (0)
Distant	40 (31.3)	5 (10.2)	15 (48.4)	19 (54.3)	0 (0)	1 (33.3)
*Localized patients at initial diagnosis*						
Recurrence/Metastasis status						
No relapse	25 (31.6)	13 (31)	3 (27.3)	4 (28.6)	5 (50)	0 (0)
Local relapse	22 (27.8)	12 (28.6)	1 (9.1)	7 (50)	0 (0)	2 (100)
Metastasis^c^	32 (40.5)	17 (40.5)	7 (63.6)	3 (21.4)	5 (50)	0 (0)
Time to first metastasis, months (mean, range)	13.2 ± 10.9 (1–44)	12.8 ± 11.9 (2–44)	11.9 ± 8.4 (4–27)	11.3 ± 13.1 (1–26)	17.4 ± 11.5 (9–37)	NA
*Metastasis patients throughout the follow-up*						
Pulmonary metastasis						
No	51 (63)	10 (41.7)	21 (77.8)	16 (66.7)	3 (60)	1 (100)
Yes	24 (29.6)	8 (33.3)	6 (22.2)	8 (33.3)	2 (40)	0 (0)
Unknown	6 (7.4)	6 (25)	0 (0)	0 (0)	0 (0)	0 (0)
Bone metastasis						
No	48 (59.3)	11 (45.8)	18 (66.7)	16 (66.7)	2 (40)	1 (100)
Yes	27 (33.3)	7 (29.2)	9 (33.3)	8 (33.3)	3 (60)	0 (0)
Unknown	6 (7.4)	6 (25)	0 (0)	0 (0)	0 (0)	0 (0)
Liver metastasis						
No	62 (76.5)	17 (70.8)	24 (88.9)	17 (70.8)	4 (80)	0 (0)
Yes	13 (16)	1 (4.2)	3 (11.1)	7 (29.2)	1 (20)	1 (100)
Unknown	6 (7.4)	6 (25)	0 (0)	0 (0)	0 (0)	0 (0)
*Survival of all patients*						
Recurrence						
No	36 (28.1)	15 (30.6)	6 (19.4)	9 (25.7)	5 (50)	1 (33.3)
Yes	92 (71.9)	34 (69.4)	25 (80.6)	26 (74.3)	5 (50)	2 (66.7)
Dead						
No	56 (43.8)	24 (49)	11 (35.5)	13 (37.1)	6 (60)	2 (66.7)
Yes	72 (56.3)	25 (51)	20 (64.5)	22 (62.9)	4 (40)	1 (33.3)
Median PFS (range), months	7 (1–190)	10 (1–190)	5 (1–81)	6 (1–88)	17 (9–37)	2 (1–2)
Median OS (range), months	20 (2–190)	37 (4–190)	11 (2–81)	12 (2–179)	34 (9–45)	3 (2–6)

AS: Angiosarcoma; NA: Not applicable; PFS: Progression-free survival; OS: Overall survival

a: Including patients with both regional lymph node involvement and distant metastasis

b: Including patients with only lymph node involvement

c: Including patients with both local relapse and distant metastasis

#### Baseline clinicopathological characteristics at initial diagnosis

In this study, most patients had cutaneous AS (*n* = 49; 38.3%), predominantly on the scalp, followed by viscera (*n* = 35; 27.3%), deep soft tissue (*n* = 31; 24.2%), breast (*n* = 10; 7.8%) and bone (*n* = 3; 2.3%). The mean age for cutaneous AS patients was 66.3 years, higher than that for other locations, while breast and bone AS patients were the youngest. Males were predominant, particularly in cutaneous AS cases. Radiation-induced secondary AS was rare (7 cases; 5.5%), occurring in deep soft tissues, viscera, and the breast, with over 5 years between radiotherapy and AS in 5 cases. The existing data showed that 18 patients had low-grade tumors and 12 had high-grade, and all breast AS cases were low-grade. Tumor necrosis was observed in 27 patients, most commonly in deep soft tissues and viscera. Viscera AS tumors (heart, liver, spleen, lung, kidney, uterus, and colon) were larger on average (7.7 cm), with 54.3% over 5 cm, more than in other sites. At initial diagnosis, 79 patients (61.7%) had localized tumors, 13 patients (10.2%) had regional lymph node metastasis, and 40 patients (31.3%) had distant metastasis. Among the 9 patients with only lymph node metastasis, 4 developed distant metastases. Distant metastasis was primarily concentrated in deep soft tissues and visceral areas. In contrast, among superficial AS patients, 85.7% were localized tumors, with only 5 cases showing distant metastasis, and all breast AS patients were localized.

#### Recurrence and metastasis overview

At the end of the follow-up, among patients initially diagnosed with localized tumors, 25 (31.6%) had no recurrence, 22 (27.8%) had local recurrence, and 32 (40.5%) developed distant metastases. Notably, 63.6% of localized deep soft tissue AS cases developed metastasis. On average, distant metastasis occurred after 13.2 months, with deep soft tissue and visceral AS showing the shortest time to metastasis (11.9 and 11.3 months), and breast AS the longest (17.4 months).

Among the 81 patients with metastases, 27 had bone metastases, 24 lung metastases, and 13 liver metastases. In cutaneous AS, metastases commonly occurred in the lungs and bones, with liver metastasis being rare. Other less common sites included distant lymph nodes, spleen, pleura, peritoneum, bladder, parotid gland, and so on.

#### Molecular characteristics and PD-L1 expression

PD-L1 expression, tested in 4 patients, showed a CPS of 20 in one case, who received late-line immunotherapy with a poor outcome, and low CPS in three cases. NGS was performed in 7 cases, revealing TMB-H with MSI-H in one case, who received first-line immunotherapy and achieved a favorable outcome, and TMB-L with MSS in three cases. Additionally, *TP53* mutation was identified in five cases, while *KDR* and *CDKN2A* mutations were found in one case each. *MYC* amplification was detected in two cases (Table S1).

#### Survival outcome of all patients

During treatment, 92 patients (71.9%) experienced recurrence, and 72 patients (56.3%) died from AS. Recurrence and mortality rates were higher in deep soft tissue and viscera AS. Among all 128 patients, the 2-year PFS was 21.4%, with a median PFS of 7 months. The 5-year OS was 29.6%, with a median OS of 20 months. Survival varied significantly by AS location; median OS for cutaneous and breast AS (37 and 34 months) was notably better than for deep soft tissue, viscera, and bone AS (11, 12, and 3 months, respectively) (*P* = 0.004). 

### Prognostic clinical factors (Table 2)

**Table 2:** Univariate and multivariate analyses of survival in patients with AS

		Progression-free survival				Overall survival			
		Univariate		Multivariate		Univariate		Multivariate	
	No	HR (95%CI)	P value	HR (95%CI)	*P* value	HR (95%CI)	*P* value	HR (95%CI)	*P* value
*At initial diagnosis*									
Age (years)									
< 60	64	1		1		1		1	
≥ 60	64	1.223 (0.806–1.857)	0.344	1.210 (0.751–1.949)	0.433	1.049 (0.660–1.667)	0.839	1.051 (0.606–1.822)	0.859
Gender									
Male	75	1		1		1		1	
Female	53	0.468 (0.301–0.726)	< **0.001**	0.580 (0.350–0.961)	**0.034**	0.681 (0.422–1.099)	0.116	0.690 (0.401–1.187)	0.180
Tumor differentiation									
Low grade	18	1		1		1		1	
High grade	12	1.509 (0.598–3.810)	0.384	1.408 (0.524–3.779)	0.497	1.924 (0.609–6.075)	0.265	2.177 (0.644–7.354)	0.211
Tumor necrosis									
No	34	1		1		1		1	
Yes	27	1.359 (0.764–2.419)	0.296	0.778 (0.403–1.502)	0.455	1.517 (0.795–2.893)	0.206	0.995 (0.493–2.008)	0.989
Anatomic site									
Cutaneous	49	1		1		1		1	
Deep soft tissue	31	1.794 (1.064–3.025)	**0.028**	2.006 (1.046–3.846)	**0.036**	2.207 (1.217–4.004)	**0.009**	2.276 (1.098–4.719)	**0.027**
Viscera	35	1.759 (1.051–2.944)	**0.032**	1.789 (0.932–3.434)	0.080	2.316 (1.298–4.131)	**0.004**	2.253 (1.128–4.500)	**0.021**
Breast	10	0.480 (0.187–1.231)	0.127	0.996 (0.343–2.891)	0.994	0.828 (0.287–2.393)	0.728	1.815 (0.530–6.223)	0.343
Bone	3	5.821 (1.321–25.644)	**0.020**	5.685 (1.202–26.889)	**0.028**	6.345 (0.820–49.092)	0.077	8.304 (0.900–76.602)	0.062
Maximum diameter of tumor, cm									
≤ 5	68	1		1		1		1	
> 5	43	1.686 (1.085–2.620)	**0.020**	1.724 (1.028–2.890)	**0.039**	2.035 (1.235–3.353)	**0.005**	1.894 (1.064–3.373)	**0.030**
Clinical stage									
Localized	79	1		1		1		1	
Regional	9	0.944 (0.375–2.375)	0.902	0.624 (0.228–1.707)	0.359	2.130 (0.817–5.550)	0.122	2.028 (0.722–5.690)	0.179
Distant^a^	40	2.205 (1.410–3.448)	< **0.001**	1.467 (0.850–2.531)	0.169	2.891 (1.742–4.798)	< **0.001**	1.992 (1.062–3.738)	**0.032**
*Primary treatment*									
Surgery									
All patients									
No	35	1		1		1		1	
Yes	93	0.386 (0.243–0.614)	< **0.001**	0.537 (0.296–0.977)	**0.042**	0.431 (0.260–0.714)	**0.001**	0.551 (0.284–1.070)	0.078
Localized patients									
No	12	1				1			
Yes	67	0.225 (0.112–0.452)	** < 0.001**			0.368 (0.173–0.786)	**0.010**		
Metastatic patients									
No	23	1				1			
Yes	26	0.802 (0.418–1.537)	0.506			0.985 (0.485–1.999)	0.966		
Surgical margin after primary surgery									
Negative/R0	77	1				1			
Positive/R1 + R2	14	1.522 (0.786–2.946)	0.213			1.578 (0.755–3.295)	0.225		
Surgery + adjuvant radiotherapy									
All patients									
No	73	1				1			
Yes	20	0.535 (0.271–1.055)	0.071			0.617 (0.275–1.382)	0.240		
Localized									
No	51	1				1			
Yes	16	0.393 (0.165–0.936)	**0.035**			0.339 (0.103–1.120)	0.076		
Surgery + adjuvant chemotherapy									
All patients									
No	50	1				1			
Yes	43	0.920 (0.555–1.523)	0.745			0.764 (0.424–1.379)	0.372		
Localized									
No	37	1				1			
Yes	30	0.853 (0.460–1.583)	0.614			0.759 (0.368–1.566)	0.456		
Radiotherapy									
No	101	1		1		1		1	
Yes	27	0.648 (0.382–1.100)	0.108	0.632 (0.354–1.127)	0.120	0.705 (0.385–1.288)	0.255	0.787 (0.410–1.509)	0.470
Chemotherapy									
No	58	1		1		1		1	
Yes	70	1.146 (0.756–1.736)	0.522	0.986 (0.613–1.586)	0.955	0.969 (0.609–1.543)	0.895	0.642 (0.364–1.134)	0.127
Targeted therapy									
No	90	1		1		1		1	
Yes	38	1.017 (0.631–1.638)	0.945	0.597 (0.323–1.104)	0.100	1.137 (0.663–1.952)	0.641	0.650 (0.316–1.336)	0.241

Bold indicates statistically significant results (*P* < 0.05)

AS: Angiosarcoma; HR: Hazard ratio; CI: Confidence intervals; R0: No macroscopic; R1: Microscopic residual lesions; R2: Macroscopic residual lesions

a: Including patients with both regional lymph node metastasis and distant metastasis

**Fig. 1:**
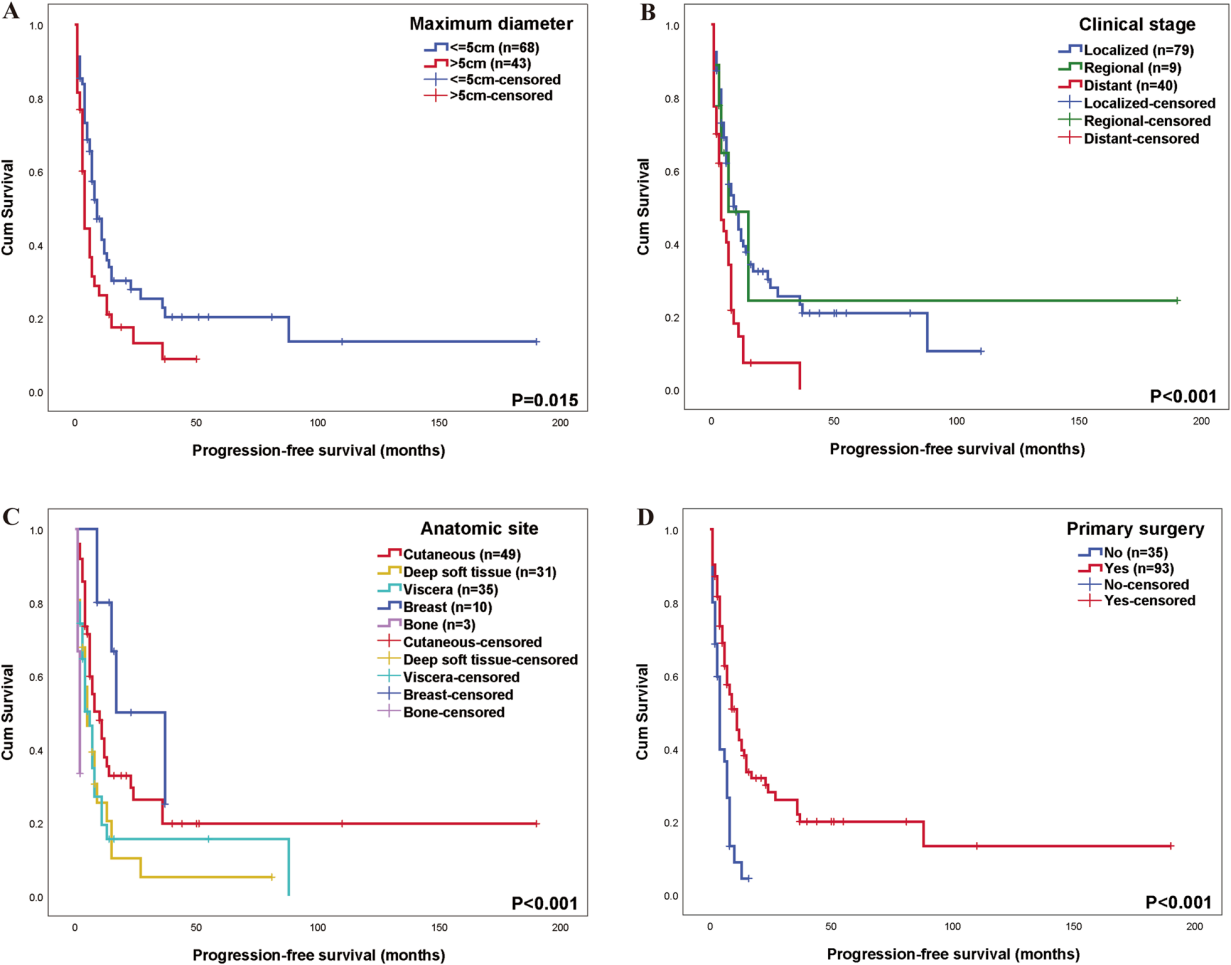
Progression-free survival. **A** Kaplan–Meier survival curves illustrating the difference between tumor size ≤ 5 cm and > 5 cm; **B** Kaplan–Meier survival curves illustrating the difference between patients with localized disease, only regional lymph node involvement and distant metastases; **C** Kaplan–Meier survival curves illustrating the difference between various anatomic sites, including cutaneous, deep soft tissue, viscera, breast and bone; **D** Kaplan–Meier survival curves illustrating the difference between whole patients with primary surgery and without

Univariate Cox regression analysis identified several clinical factors significantly associated with PFS. Female gender was linked to improved PFS (HR = 0.468, *P* < 0.001), while tumor size > 5 cm (HR = 1.686, *P* = 0.020) and distant metastasis (HR = 2.205, *P* < 0.001) were associated with worse PFS. Tumor location also impacted PFS, compared to cutaneous tumors, those in deep soft tissue (HR = 1.794, *P* = 0.028), viscera (HR = 1.759, *P* = 0.032), and bone (HR = 5.821, *P* = 0.020) showed poorer PFS (Fig. 1A, B, C). All these variables were included in the multivariate Cox regression analysis. The results demonstrated that gender, tumor size, and anatomic site remained independent prognostic factors for PFS.

**Fig. 2:**
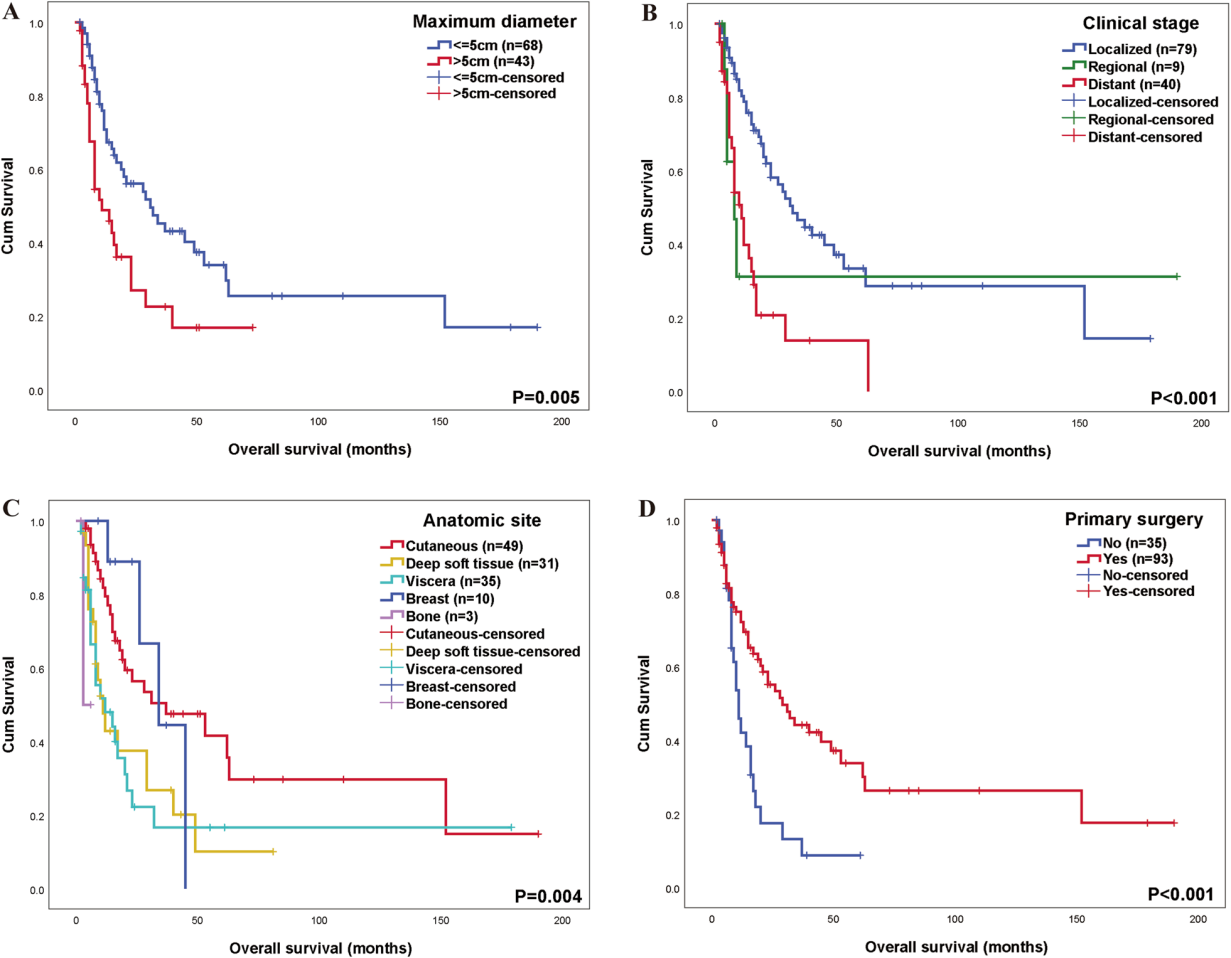
Overall survival. **A** Kaplan–Meier survival curves illustrating the difference between tumor size ≤ 5 cm and > 5 cm; **B** Kaplan–Meier survival curves illustrating the difference between patients with localized disease, only regional lymph node involvement and distant metastases; **C** Kaplan–Meier survival curves illustrating the difference between various anatomic sites, including cutaneous, deep soft tissue, viscera, breast and bone; **D** Kaplan–Meier survival curves illustrating the difference between whole patients with primary surgery and without

Both univariate and multivariate Cox regression analyses demonstrated that tumor size > 5 cm (HR = 2.035, *P* = 0.005), clinical stage (distant) (HR = 2.891, *P* < 0.001), and anatomic site (deep soft tissue vs. cutaneous, HR = 2.207, *P* = 0.009; viscera vs. cutaneous, HR = 2.316, *P* = 0.004) were significantly associated with poor OS (Fig. 2A, B, C).

### Primary treatment (Table 2)

#### Surgery

A total of 93 patients (72.7%) underwent primary surgical resection. It was significantly associated with improved PFS (HR = 0.386, *P* < 0.001) (Fig. 1D) and OS (HR = 0.431, *P* = 0.001) (Fig. 2D).

In patients with localized disease (*n* = 67), surgery conferred significant survival benefits for both PFS (HR = 0.225, *P* < 0.001) and OS (HR = 0.368, *P* = 0.010). No survival benefit was observed in the metastatic subgroup. Regarding surgical margins, most patients achieved R0 resection (*n* = 77), and margin status (R0 vs. R1/R2) was not significantly associated with survival.

#### Radiotherapy

Radiotherapy was administered to 27 patients (20.9%) during primary treatment, but its use was not significantly associated with survival. The median radiation dose administered as part of primary treatment was 60 Gy (range: 45–70 Gy), while for adjuvant therapy, the median dose was 50 Gy (range: 30–60 Gy). Patients with cutaneous angiosarcoma (AS) most frequently received radiotherapy, suggesting a potentially greater role of radiotherapy in this subgroup.

Notably, for localized disease, combined primary surgery with adjuvant radiotherapy showed a significant improvement in PFS (HR = 0.393, *P* = 0.035), but had no effect on OS (HR = 0.339, *P* = 0.076).

#### Chemotherapy

A total of 70 patients (53.0%) received chemotherapy in the initial treatment phase. No significant association was found between chemotherapy and survival outcomes. Moreover, adjuvant chemotherapy did not enhance the efficacy of primary surgery.

### Treatment for metastatic or unresectable AS and outcomes (Table 3)

**Table 3:** Treatment for AS with advanced/metastatic disease from different anatomical sites

	All patients (N%)	Cutaneous (N%)	Deep soft tissue (N%)	Viscera (N%)	Breast (N%)	Bone (N%)
Total	81 (100)	24 (29.6)	27 (33.3)	24 (29.6)	5 (6.2)	1 (1.2)
Surgery						
No	50 (61.7)	16 (66.7)	16 (59.3)	13 (54.2)	5 (100)	0 (0)
Yes	31 (38.3)	8 (33.3)	11 (40.7)	11 (45.8)	0 (0)	1 (100)
Radiotherapy						
No	64 (79)	19 (79.2)	20 (74.1)	19 (79.2)	5 (100)	1 (100)
Yes	17 (21)	5 (20.8)	7 (25.9)	5 (20.8)	0 (0)	0 (0)
Supportive care only						
No	68 (84)	14 (58.3)	25 (92.6)	24 (100)	4 (80)	1 (100)
Yes	13 (16)	10 (41.7)	2 (7.4)	0 (0)	1 (20)	0 (0)
Prior chemotherapy						
No	17 (53.1)	8 (47.1)	4 (57.1)	1 (33.3)	4 (80)	1 (100)
Paclitaxel-based	7 (21.9)	4 (23.5)	1 (14.3)	1 (33.3)	1 (20)	0 (0)
Doxorubicin-based	4 (12.5)	2 (11.8)	2 (28.6)	0 (0)	0 (0)	0 (0)
Paclitaxel and doxorubicin	4 (12.5)	3 (17.6)	0 (0)	1 (33.3)	0 (0)	0 (0)
*First-line treatment (n* = *49)*						
Strategy						
Chemotherapy only	19 (38.8)	6 (75)	5 (27.8)	5 (26.3)	3 (75)	NA
Chemotherapy + Targeted therapy^a^	21 (42.9)	2 (25)	11 (61.1)	7 (36.8)	1 (25)	NA
Chemotherapy + Immunotherapy^b^	2 (4.1)	0 (0)	0 (0)	2 (10.5)	0 (0)	NA
Chemotherapy + Targeted therapy + Immunotherapy	4 (8.2)	0 (0)	1 (5.6)	3 (15.8)	0 (0)	NA
Targeted therapy + Immunotherapy	1 (2)	0 (0)	1 (5.6)	0 (0)	0 (0)	NA
Targeted therapy only	2 (4.1)	0 (0)	0 (0)	2 (10.5)	0 (0)	NA
Chemotherapy regimen						
Paclitaxel-based	32 (71.1)	6 (75)	14 (82.4)	10 (62.5)	2 (50)	NA
Doxorubicin-based	13 (28.9)	2 (25)	3 (17.6)	6 (37.5)	2 (50)	NA
Therapeutic evaluation after first treatment						
PR	16 (32.7)	6 (75)	4 (22.2)	5 (26.3)	1 (25)	NA
SD	18 (36.7)	1 (12.5)	8 (44.4)	7 (36.8)	2 (50)	NA
PD	15 (30.6)	1 (12.5)	6 (33.3)	7 (36.8)	1 (25)	NA
PFS1: months (median, range)	5 (1–16)	6 (2–13)	5 (1–13)	4 (1–16)	4 (3–5)	NA
*Second-line treatment (n* = *18)*						
Strategy						
Chemotherapy only	3 (16.7)	0 (0)	1 (14.3)	2 (28.6)	0 (0)	NA
Chemotherapy + Targeted therapy^a^	6 (33.3)	0 (0)	3 (42.9)	3 (42.9)	0 (0)	NA
Chemotherapy + Immunotherapy^b^	1 (5.6)	1 (50)	0 (0)	0 (0)	0 (0)	NA
Chemotherapy + Targeted therapy + Immunotherapy	3 (16.7)	0 (0)	1 (14.3)	1 (14.3)	1 (50)	NA
Targeted therapy + Immunotherapy	1 (5.6)	0 (0)	0 (0)	1 (14.3)	0 (0)	NA
Targeted therapy only	4 (22.2)	1 (50)	2 (28.6)	0 (0)	1 (50)	NA
Chemotherapy regimen						
Paclitaxel-based	6 (50)	1 (100)	3 (60)	2 (40)	0 (0)	NA
Doxorubicin-based	6 (50)	0 (0)	2 (40)	3 (60)	1 (100)	NA
Therapeutic evaluation after second treatment						
PR	3 (16.7)	0 (0)	2 (28.6)	1 (14.3)	0 (0)	NA
SD	2 (11.1)	0 (0)	0 (0)	1 (14.3)	1 (50)	NA
PD	13 (72.2)	2 (100)	5 (71.4)	5 (71.4)	1 (50)	NA
PFS2: months (median, range)	2 (1–17)	1 (1–2)	2 (1–17)	2 (1–6)	1 (1–3)	NA
*Third-line treatment (n* = *10)*						
Strategy						
Chemotherapy only	1 (10)	0 (0)	1 (25)	0 (0)	0 (0)	NA
Chemotherapy + Targeted therapy^a^	3 (30)	0 (0)	1 (25)	1 (33.3)	1 (100)	NA
Chemotherapy + Immunotherapy^b^	3 (30)	2 (100)	0 (0)	1 (33.3)	0 (0)	NA
Chemotherapy + Targeted therapy + Immunotherapy	1 (10)	0 (0)	1 (25)	0 (0)	0 (0)	NA
Targeted therapy + Immunotherapy	1 (10)	0 (0)	1 (25)	0 (0)	0 (0)	NA
Targeted therapy only	1 (10)	0 (0)	0 (0)	1 (33.3)	0 (0)	NA
Chemotherapy regimen						
Paclitaxel-based	2 (40)	0 (0)	1 (33.3)	1 (100)	NA	NA
Doxorubicin-based	3 (60)	1 (100)	2 (66.7)	0 (0)	NA	NA
Therapeutic evaluation after second treatment						
PR	2 (20)	0 (0)	1 (25)	1 (33.3)	0 (0)	NA
SD	3 (30)	1 (50)	1 (25)	0 (0)	1 (100)	NA
PD	5 (50)	1 (50)	2 (50)	2 (66.7)	0 (0)	NA
PFS3: months (median, range)	2 (1–5)	1 (1–3)	2 (1–3)	2 (1–5)	4	NA
Death						
No	21 (25.9)	4 (16.7)	8 (29.6)	7 (29.2)	1 (20)	1 (100)
Yes	60 (74.1)	20 (83.3)	19 (70.4)	17 (70.8)	4 (80)	0 (0)
OS: months (median, range)^c^	9 (1–190)	9 (1–190)	11 (1–39)	8 (2–24)	8 (4–20)	NA

AS: Angiosarcoma; CR: Complete response; PR: Partial response; SD: Stable disease; PD: Progressive disease; PFS: Progression-free survival; OS: Overall survival; NA: Not applicable

a: Totally are anti-angiogenic drugs, including bevacizumab and TKIs (tyrosine kinase inhibitors)

b: Totally are immune checkpoint inhibitors specifically target PD-1 (programmed cell death protein-1)

c: Overall survival calculated from the onset of metastasis

This table summarized palliative therapies and outcomes for 81 advanced/metastatic AS from different anatomical sites. Surgery for local recurrence or metastatic lesions was performed in 38.3% of cases. Palliative radiotherapy was administered to 21% of patients, with a median dose of 30 Gy (range 8–66). Some patients opted for transarterial chemoembolization (TACE) for liver metastases, which reduced tumor size but had a short duration of control. Additionally, 16% of patients only received supportive care, resulting in poor outcome with a median OS of just 2 months (rang 1–14).

Chemotherapy was the primary treatment, with paclitaxel-based regimens (mainly nab-paclitaxel monotherapy or combined with platinum derivatives) and doxorubicin-based regimens (mostly combined with ifosfamide) as the main strategies. Additional options included temozolomide, dacarbazine, gemcitabine and eribulin. Anti-angiogenic therapies included bevacizumab and TKIs such as lenvatinib, sorafenib, anlotinib, and apatinib. Immunotherapy involved PD-1 inhibitors like pembrolizumab, sintilimab, and tislelizumab.

A high mortality rate of 74.1% was noted during follow-up, with a median OS from the onset of metastasis of 9 months. No significant survival difference was observed among patients from different anatomical sites.

#### First-line treatment

Most patients (93.9%) received chemotherapy, 57.1% received targeted therapy, while only 13.4% patients received immunotherapy. None of the cutaneous or breast AS patients received targeted therapy or immunotherapy. Overall, the ORR after first-line treatment was 32.7%, with notable 30.6% experiencing disease progression. The median PFS1 was 5 months. Cutaneous AS showed the best treatment outcomes, followed by breast AS. The poorest outcomes were seen in deep soft tissue and viscera AS, with DCRs of 87.5%, 75%, 66.7%, and 63.3%, respectively.

**Fig. 3:**
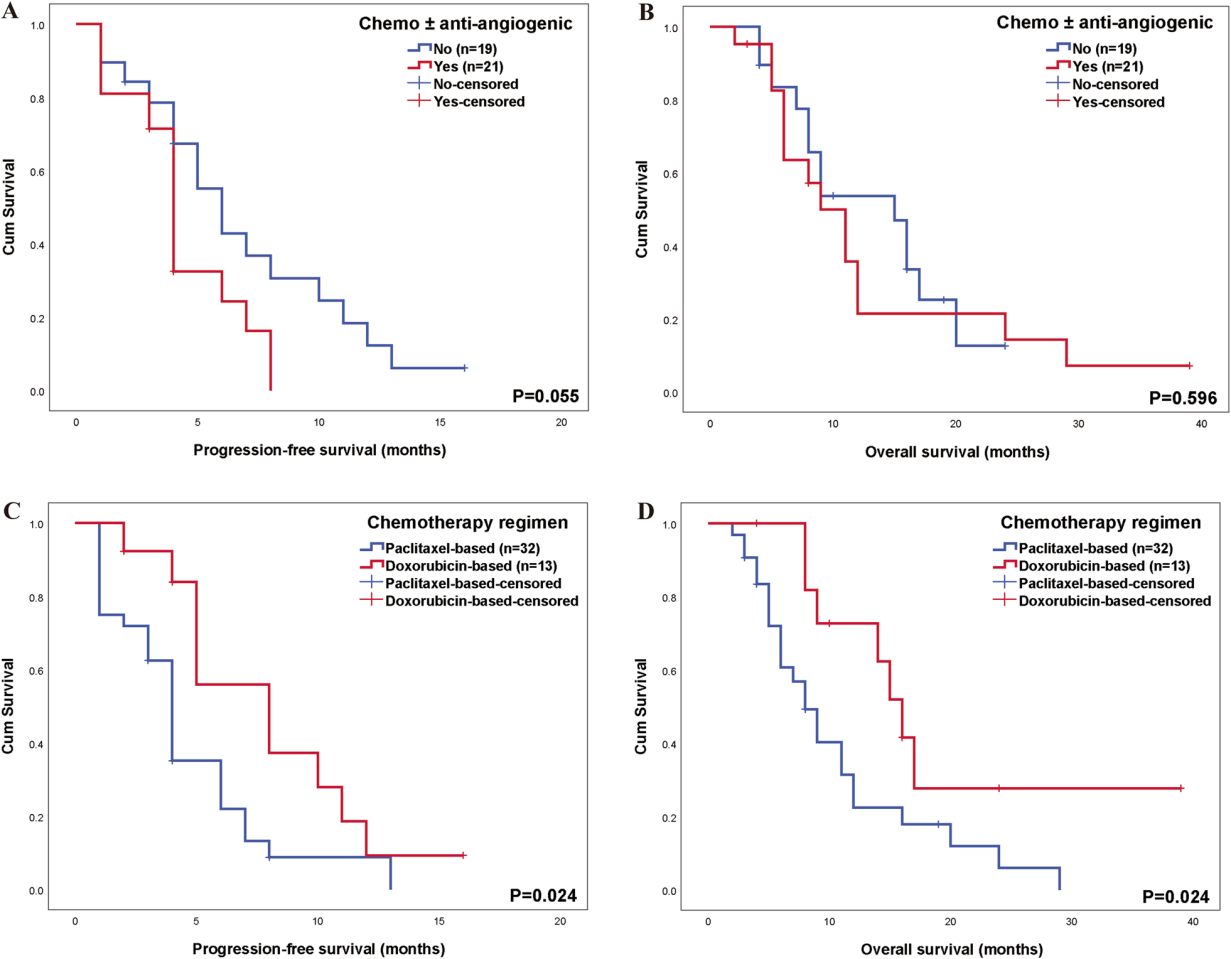
First-line treatment for metastatic angiosarcoma patients. **A** Comparison of progression-free survival between chemotherapy alone and chemotherapy combined with anti-angiogenic therapy; **B** Comparison of overall survival between chemotherapy alone and chemotherapy combined with anti-angiogenic therapy; **C** Comparison of progression-free survival between paclitaxel-based and doxorubicin-based chemotherapy; **D** Comparison of overall survival between paclitaxel-based and doxorubicin-based chemotherapy

1. Chemotherapy combined with anti-angiogenic therapy did not show superior survival outcomes compared to chemotherapy alone. In the chemotherapy-only group (with a similar distribution of paclitaxel- and doxorubicin-based regimens), the ORR was 47.4% (9/19), and the DCR was 73.7% (14/19). In the group receiving chemotherapy (mainly paclitaxel-based) combined with anti-angiogenic therapy (primarily bevacizumab), the ORR was lower at 28.6% (6/21), while the DCR was similar at 71.4% (15/21). Both groups had 6 patients who did not complete all chemotherapy cycles. Kaplan–Meier analysis confirmed these findings, showing a median PFS of 4 months for combination therapy versus 6 months for chemotherapy alone (*P* = 0.055), and a median OS of 9 months vs. 15 months (*P* = 0.596) (Fig. 3A, B).
2. The combination of chemotherapy and immunotherapy (3 cases with pembrolizumab, 2 with sintilimab, and 1 with toripalimab) showed limited efficacy, with 2 cases of SD and 4 of PD. Notably, 4 patients did not complete the chemotherapy cycles, and 1 patient received only oral temozolomide.
3. Among the 3 patients who did not receive systemic chemotherapy, 2 were treated with oral sorafenib alone, both achieving SD. One patient received apatinib and PD-1 inhibitor, plus metastasis reduction surgery and local palliative radiotherapy, achieving PR and a PFS of 13 months.
4. For first-line chemotherapy, 32 patients received paclitaxel-based regimens (2 had prior doxorubicin treatment), and 13 patients received doxorubicin-based regimens (3 had prior paclitaxel treatment). The mean ages were similar: 53.53 years for the paclitaxel group and 52.46 years for the doxorubicin group. Completion rates of chemotherapy cycles were comparable at 62.5% vs. 69.2%. The paclitaxel group more frequently combined with targeted therapy but showed worse outcomes compared to doxorubicin group: ORR was 28.1% vs. 46.2%, DCR was 62.5% vs. 84.6%. Median PFS was 4 vs. 8 months (*P* = 0.024), and median OS was 8 vs. 16 months (*P* = 0.024) from the start of chemotherapy (Fig. 3C, D).

#### Later-line treatment

Similar patterns were observed in the second- and third-line treatments, with chemotherapy-based regimens being most common. More patients received targeted and immunotherapy, but the overall treatment outcomes were suboptimal, with disease progression in over half of the cases. Median PFS for both second- and third-line treatments was 2 months. Switching between paclitaxel- and doxorubicin-based regimens was common across treatment lines. Eribulin and gemcitabine were always used in later-line chemotherapy.

## Discussion

This retrospective study of 128 AS patients across various anatomical sites highlights the heterogeneity in clinical presentation, treatment response, and survival outcomes, providing valuable insights for clinical practice and future research.

Cutaneous AS, especially on the scalp, was the most common, with older, predominantly male patients. Breast and bone AS occurred in younger individuals. Superficial AS was often localized, while deep-seated AS had larger tumors and a higher risk of early distant metastases, often occurring within the first year of diagnosis, reflecting greater aggressiveness. Survival outcomes mirrored this, with superficial AS showing significantly longer PFS and OS. However, survival data for bone AS may be less reliable due to the small sample size of three cases.

Previous studies identified primary AS, age, tumor grade, tumor size and sites as key prognostic factors [16]. Cutaneous AS had a significantly lower risk of death than visceral AS (HR = 0.63) [33]. Our data also showed poorer prognoses with larger tumors, distant metastases, and deeper anatomical involvement. Deep soft tissue AS had a higher mortality risk than cutaneous AS (HR = 2.207), with viscera AS showing an even greater risk (HR = 2.316). In our study, radiation-induced AS, seen in only seven cases (5.5%), limited comparisons with primary AS. Additionally, a prior study reported worse outcomes with liver metastases [22]. We found no prognostic impact of liver metastases, but they were more common in deep AS.

### Primary surgery and adjuvant therapy

Primary surgery improved both PFS and OS for AS patients, particularly those with localized disease, consistent with prior studies [16]. However, no benefit was seen in metastatic cases.

The survival benefit of R0 resection remains debated [16]. Our findings indicated that positive surgical margin (R1/R2) did not significantly affect prognosis, but macroscopic residual lesions (R2) markedly increased recurrence risk (HR 5.725, 95% CI [1.259, 10.110], *P* = 0.017).

Given the high postoperative local recurrence rate in AS, adjuvant radiotherapy (50–70 Gy) is often administered to improve local control. In this study, 20 patients received adjuvant radiotherapy (median dose 50 Gy), improving PFS in localized cases, but not in all patients or OS. Adjuvant chemotherapy, administered to 43 patients, showed no substantial impact on improving prognosis, aligning with previous studies [17, 18].

### Treatment for advanced/metastatic AS

Treatment outcomes of metastatic AS were generally poor, with a median OS of just 9 months. After first-line treatment, superficial AS showed a 75% PR rate, significantly better than deep-seated AS.

#### Chemotherapy

Key findings on chemotherapy from previous studies and our research are summarized in Table S2.

##### Paclitaxzel-based or doxorubicin-based

Unlike other STS, AS shows higher sensitivity to taxanes [34]. Both taxane- and anthracycline-based chemotherapies show activity in AS, but the optimal sequence of these regimens remains unclear.

Some retrospective studies suggest paclitaxel is more effective. In 117 metastatic AS patients, first-line paclitaxel had higher ORR (53% vs. 29%) and better OS (10.3 vs. 5.5 months) than doxorubicin, with no PFS difference. Notably, more cutaneous cases were in the paclitaxel group [35]. Another study found slightly better ORR for paclitaxel (45.5% vs. 30.9%), but no survival difference [36]. A 2021 Asian Sarcoma Consortium study (276 patients) showed a trend toward greater paclitaxel use and slightly better PFS (4.5 vs. 2.8 months) and OS (11.9 vs. 10.6 months) compared to liposomal doxorubicin, but no statistical significance [33].

Other studies report similar efficacy between the two regimens [37]. The ANGIOTAX trial (30 unresectable AS) treated with weekly single-agent paclitaxel showed an mPFS of 4 months and mOS of 8 months [38], identical to our findings. Similar to the outcomes of paclitaxel, a pooled analysis of 11 EORTC trials showed that 108 metastatic AS patients treated with doxorubicin had a mPFS of 4.9 months and a mOS of 9.9 months [39]. Currently, no prospective studies have directly compared the two regimens.

In our study, doxorubicin-based regimens outperformed paclitaxel-based regimens in ORR (46.2% vs. 28.1%), DCR (84.6% vs. 62.5%), mPFS (8 vs. 4 months), and mOS (16 vs. 8 months). Both groups had similar baseline characteristics, including prior exposure to the cytotoxic agents, mean age, tumor site distribution, and chemotherapy completion rates, minimizing the potential influence of confounding factors on treatment outcomes. However, the smaller doxorubicin group warrants cautious interpretation. Larger or prospective studies are needed.

##### Anthracycline monotherapy or combination with ifosfamide

The pooled analysis of 11 EORTC trials also showed combination regimens improved PFS and OS compared to doxorubicin monotherapy [39]. A phase III randomized controlled trial (EORTC 62012) demonstrated combination with ifosfamide improved ORR and PFS compared to single-agent anthracycline in STS, only 22 (4.8%) AS cases were included [40]. In our cohort, the number of patients receiving doxorubicin monotherapy was insufficient to allow for a meaningful comparison.

##### Doxorubicin or pegylated liposomal doxorubicin (PLD)

PLD offers reduced cardiac and gastrointestinal toxicity with similar efficacy to doxorubicin. A retrospective analysis (11 patients) treated with PLD showed an mPFS of 4.2 months [41], consistent with our findings. As most of our patients received PLD, no relevant comparison was made.

In addition, although small retrospective studies suggest gemcitabine efficacy in AS [42], the phase III GeDDiS trial found no advantage of gemcitabine plus docetaxel over doxorubicin as first-line treatment [43]. Given the high toxicity and poor tolerance, gemcitabine is rarely used first-line. In this study, it was reserved for later lines, like the new agent eribulin.

#### Anti-angiogenic targeted therapy

##### Anti-VEGF monoclonal antibody (bevacizumab)

Elevated VEGF and receptors expression support the use of bevacizumab in AS, and it is often combined with chemotherapy in clinical practice.

However, adding anti-angiogenic therapy has not significantly improved survival outcomes. The ANGIO-TAX-PLUS trial of 50 advanced AS patients showed paclitaxel monotherapy had higher ORR (45.8% vs. 28.0%) and longer mOS (19.5 vs. 15.9 months) than paclitaxel plus bevacizumab [44]. Similarly, in our 40 advanced AS patients, chemotherapy alone also showed a higher ORR (47.4% vs. 28.6%), longer mPFS (6 vs. 4 months) and mOS (15 vs. 9 months) than combining with anti-angiogenic therapy, though differences were not significant.

##### TKIs

In addition to bevacizumab, TKIs targeting BRAF, VEGFR, and PDGFR are used for AS treatment. A phase II trial reported an mPFS of 1.8 months for superficial and 3.8 months for viscera AS with sorafenib [45]. Pazopanib showed potential in delaying progression in taxane-resistant cutaneous AS, with an mPFS of 94 days, though based on a small cohort of 5 patients (2 PR, 2 SD, 1 PD) [46]. Case reports and small studies also suggested efficacy of sunitinib, anlotinib, apatinib [47–49], and regorafenib [50]. In our study, two patients achieved SD with first-line sorafenib. After first-line failure, 4 patients received TKI monotherapy (1 apatinib, 1 lenvatinib, 2 anlotinib), whereas none achieved disease control. Combining with PD-1 inhibitors improved efficacy in early lines (1 PR, 1 SD) but failed in third line. Although TKIs offer benefit, tumor control is limited. And most clinical data are derived from small AS subsets in sarcoma trials or retrospective studies, with lack of large-scale AS-specific research.

Endoglin, a key angiogenic target distinct from VEGFR, is upregulated in AS tumor cells following VEGFR inhibition. However, TRC105 (anti-endoglin) combined with pazopanib did not improve PFS significantly over pazopanib alone in advanced AS [51]. Ongoing trials exploring therapies like pazopanib, regorafenib, propranolol plus cyclophosphamide, durvalumab plus tremelimumab, and TRC105 plus pazopanib are awaited. A comparative overview of findings from prior literatures and the current study is presented in Table S3.

#### Immunotherapy

PD-1/PD-L1 inhibitors show potential in STS. A study of 24 AS patients reported 66% were PD-L1 positive, with visceral tumors showing the lowest expression. High-grade tumors had higher PD-L1 expression than low-grade tumors (91.7% vs. 41.7%) [52]. In our study, only 4 patients underwent PD-L1 testing, one deep soft tissue AS showed high expression but poor response to later-line immunotherapy. Retrospective studies and case reports have confirmed the efficacy of pembrolizumab and CTLA-4 inhibitors in AS [23, 53], with notable effects in cutaneous AS patients [54]. Genetic analysis of 42 AS patients identified *TP53*, *KDR*, and *PIK3CA* as common mutations, and highest TMB in cutaneous AS [55]. High TMB may underlie immunotherapy response, one of our four tested patients with high TMB and MSI-H responded well to first-line immunotherapy. However, the ANGIOCHECK study reported higher response rates to nivolumab monotherapy in non-TMB-high cutaneous AS than TMB-high patients (28.6% vs. 14.3%) [56].

Several trials are exploring PD-1/PD-L1 inhibitors combined with other ICIs, chemotherapy, targeted therapies, and oncolytic virus treatments for AS. Ipilimumab plus nivolumab in 16 metastatic AS patients (NCT02834013) achieved a 25% ORR, with a notable 60% ORR in cutaneous AS [57]. Durvalumab plus tremelimumab in 5 patients (NCT02815995) reported a 20% ORR [58]. Paclitaxel plus avelumab as first-line treatment for unresectable AS (NCT03512834) showed a 50% ORR, a PFS of 6 months, and an OS of 14.5 months [59]. In the ongoing A091902 trial, paclitaxel plus nivolumab showed a PFS of 7.2 months in taxane-naïve AS, comparable to paclitaxel alone, but achieved a PFS of 16 months in scalp/face AS, highlighting immunotherapy's potential in cutaneous AS [60]. In our study, chemotherapy combined with immunotherapy showed suboptimal efficacy in six deep-seated AS patients (four progressed). Among these, 4 received paclitaxel, 1 received doxorubicin, and 1 was treated only with oral temozolomide. Noted that the small sample size and incomplete chemotherapy in 4 patients may have influenced the poor outcomes. Key comparative findings are summarized in Table S4.

This study has limitations inherent to its retrospective design, including potential selection and reporting biases. Additionally, the small sample size, particularly in targeted and immunotherapy subgroups, as well as the limited number of molecularly characterized cases, restricts the generalizability of these findings. Prospective studies with larger cohorts and biomarker-driven approaches are needed to validate and extend these results.

## Conclusions

This study provides a comprehensive analysis of AS across different anatomical sites, highlighting its heterogeneity as a rare malignancy. For localized AS, surgery is the standard treatment, with postoperative radiation considered to control local recurrence. For advanced AS, chemotherapy remains the cornerstone, while current treatments offer limited efficacy. Emerging targeted therapies and immunotherapies show promise, the identification of molecular characteristics lays the groundwork for personalized and more effective therapies. The combination of ICIs with other treatments may represent a future direction for AS management, particularly for high-risk subtypes like deep seated AS or later line therapy, and more clinical trials are needed for validation.

## Supplementary Information

Supplementary Material 1.

Supplementary Material 2.

Supplementary Material 3.

Supplementary Material 4.

## Data Availability

The related data were available from the corresponding author upon reasonable request.
