# Supplementary material for: Clinicopathological features, treatment outcomes, and prognostic factors of angiosarcoma: a 21-year experience at one center
Source: Orphanet J Rare Dis. 2025 Jun 11;20:298. doi: 10.1186/s13023-025-03819-9 (PMC12153173; doi:10.1186/s13023-025-03819-9)
Supplement: Supplementary file 1 — Supplementary Material 1. [file 13023_2025_3819_MOESM1_ESM.docx]

| **Table S1 Molecular characteristics and PD-L1 expression of 9 patients with AS** | | | | | | | |
| --- | --- | --- | --- | --- | --- | --- | --- |
| **Case ID** | **Anatomic site** | **TMB (mut/Mb)** | **Microsatellite status** | **PD-L1 (CPS)** | **Genetic variations** | **Immunotherapy** | **Therapeutic evaluation** |
| P01 | Visceral (Heart) | NA | NA | 2 | *ARIDIA/NOTCHI/KDR* mutations | Later-line | PD |
| P02 | Deep soft tissue | 9.76 | MSI-H | NA | *TP53* mutation | First-line | SD (PFS: 8 months) |
| P03 | Cutaneous | 2.88 | MSS | NA | *ATM/TP53/NOTCH2* mutations *MDM4/AKT1* amplifications | NA | NA |
| P04 | Visceral (Uterus) | 6.65 | MSS | NA | *TP53/CDKN2A* mutations *BRCA2/FANCA/POLD1* copy number deletions | First-line | PD |
| P05 | Deep soft tissue | NA | MSS | 20 | NA | Later-line | PD |
| P06 | Deep soft tissue | 5.76 | MSS | NA | *BRCA1/TP53* mutations *CCND2/MYCL1/AKT3* amplifications | NA | NA |
| P07 | Cutaneous | NA | NA | 3 | NA | Later-line | SD (PFS: 3 months) |
| P08 | Deep soft tissue | NA | NA | 3 | NA | NA | NA |
| P09 | Bone | NA | NA | NA | *TP53* mutation *MYC* amplification | NA | NA |
| *AS, Angiosarcoma; TMB, Tumor mutational burden; mut/Mb, mutations per megabase; MSI-H, Microsatellite instability-high; MSS, Microsatellite stable; PD-L1, Programmed death-ligand 1; CPS, Combined positive score; NA, Not applicable; PD, Progressive disease; SD, Stable disease; PFS, Progression-free survival* | | | | | | | |
