# Supplementary material for: Clinicopathological features, treatment outcomes, and prognostic factors of angiosarcoma: a 21-year experience at one center
Source: Orphanet J Rare Dis. 2025 Jun 11;20:298. doi: 10.1186/s13023-025-03819-9 (PMC12153173; doi:10.1186/s13023-025-03819-9)
Supplement: Supplementary file 2 — Supplementary Material 2. [file 13023_2025_3819_MOESM2_ESM.docx]

| **Table S2 Summary of key findings on chemotherapy for metastatic AS** | | | | | | |
| --- | --- | --- | --- | --- | --- | --- |
| **Author/Year** | **Patients** | **No.** | **Regimen** | **ORR** | **Survival** | **Key Findings** |
| Italiano A et al. ^(35)^, 2012 | Metastatic AS (cutaneous/extracutaneous) | 117 | Paclitaxel vs. Doxorubicin | 53% vs. 29% (P = 0.02) | mPFS: 5.8 vs. 3 mo (P>0.05) mOS: 10.3 vs. 5.5 mo (P = 0.002) | Paclitaxel superior in ORR and OS; more cutaneous AS in paclitaxel group |
| Penel N *et al.* ^(36)^, 2012 | Metastatic AS (superficial/visceral/bone) | 117 | Paclitaxel vs. Doxorubicin-based | 45.5% vs. 30.9% | mPFS: 5.6 vs. 5.9 mo mOS: 13.1 vs. 11 mo | Slightly better ORR for paclitaxel;  PFS and OS between the two groups are similar |
| Chen TW *et al.* ^(33)^, 2021 | Advanced AS (cutaneous/visceral) | 276 | Paclitaxel vs. Liposomal Doxorubicin | — | mPFS: 4.5 vs. 2.8 mo (P = 0.8) mOS: 11.9 vs. 10.6 mo (P = 0.5) | Trend favoring paclitaxel, not statistically significant |
| D'Angelo SP et al. ^(37)^, 2015 | Metastatic AS | 119 | Taxane-based vs. Anthracycline-based | — | mOS: 11.6 vs. 12 mo | OS between the two groups are similar |
| Penel N et al. (ANGIOTAX trial) ^(38)^, 2008 | Unresectable/metastatic AS | 30 | Weekly paclitaxel | 18% | mPFS: 4 mo mOS: 8 mo | Comparable to current study outcomes |
| Young RJ et al. ^(39)^, 2014 | Locally advanced/metastatic AS | 108 | Anthracycline-based | 25% | mPFS: 4.9 mo mOS: 9.9 mo | Similar to paclitaxel results; Doxorubicin + Ifosfamide improved PFS, and OS compared to doxorubicin monotherapy |
| Young RJ et al. ^(39)^, 2014 | Locally advanced/metastatic AS | 108 | Doxorubicin + Ifosfamide vs. Doxorubicin | — | — | Doxorubicin + Ifosfamide improved PFS and OS compared to doxorubicin monotherapy |
| Fury MG et al. ^(41)^, 2005 | Unresectable AS | 71* | Paclitaxel vs. Liposomal Doxorubicin vs.Doxorubicin vs. MAI | — | mPFS: 4.0 vs. 4.2 vs. 3.7 vs. 5.4 mo | Comparable PFS to our findings |
| Seddon B et al. (GeDDiS trial) ^(43)^, 2017 | Locally advanced/metastatic STS | 257 | Gemcitabine + Docetaxel vs. Doxorubicin | — | mPFS: 23.7 vs.23.3 weeks | No advantage for Gem + Doc over doxorubicin |
| Current study | Unresectable/metastatic AS | 45 | Doxorubicin-based vs. Paclitaxel-based | 46.2% vs. 28.1% | mPFS: 8 vs. 4 mo mOS: 16 vs. 8 mo | Doxorubicin-based regimens showed better outcomes compared to paclitaxel-based regimens |
| AS, Angiosarcoma; ORR, Objective response rate; mPFS, median progression free survival; mOS, median overall survival; MAI, Mesna + Doxorubicin + Ifosfamide; STS, Soft tissue sarcoma * Extract partial data from the study. | | | | | | |
