# Supplementary material for: Clinicopathological features, treatment outcomes, and prognostic factors of angiosarcoma: a 21-year experience at one center
Source: Orphanet J Rare Dis. 2025 Jun 11;20:298. doi: 10.1186/s13023-025-03819-9 (PMC12153173; doi:10.1186/s13023-025-03819-9)
Supplement: Supplementary file 3 — Supplementary Material 3. [file 13023_2025_3819_MOESM3_ESM.docx]

| **Table S3 Summary of key findings on targeted therapies for metastatic AS** | | | | | |
| --- | --- | --- | --- | --- | --- |
| **Study/Year** | **Patients** | **Treatment** | **No.** | **Key Findings** | **Notes** |
| ANGIO-TAX-PLUS ^(44)^, 2015 | Advanced/metastatic AS | Bevacizumab + Paclitaxel vs. Paclitaxel | 50 | ORR (28.0% vs. 45.8%); mPFS: 6.6 mo; mOS: 15.9 vs. 19.5 mo | Paclitaxel alone showed higher ORR |
| NCT00874874 ^(45)^, 2012 | Advanced/metastatic AS Superficial vs. Visceral | Sorafenib | 41 | ORR: 23%; mPFS: 1.8 vs. 3.8 mo; mOS: 12 vs. 9 mo | Tumor control duration of TKI was short |
| Ogata D et al. ^(46)^, 2016 | Taxane-resistant cutaneous AS | Pazopanib | 5 | PR=2, SD=2, PD=1; mPFS: 94 days | TKI slowed the progression of disease |
| TAPPAS ^(51)^, 2022 | Advanced AS | TRC105 (anti-Endoglin) + Pazopanib vs. Pazopanib | 114 | mPFS: 4.2 vs. 4.3 mo (P=0.95); mOS: 10.9 vs. 7.7 mo (P=0.47) | Anti-endoglin antibody combined with pazopanib did not improve PFS |
| Current study | Metastatic/unresectable AS | Anti-angiogenic therapy + Chemotherapy  vs. Chemotherapy (1st-line) | 40 | ORR: 28.6% vs. 47.4%; mPFS: 4 vs. 6 mo (P=0.055); mOS: 9 vs. 15 mo (P=0.596) | Chemotheraqpy alone showed better outcomes, alined to previous study |
| Current study | Metastatic/unresectable AS | TKIs ± PD-1 inhibitors | 8 | 2 sorafenib monotherapy (SD), 1 patinib + PD-1 inhibitor (PR) (1st-line);  4 TKIs monotherapy (PD), 1 TKI + PD-1 inhibitor (SD)(later-line) | Targeted therapies and immunotherapies offer novel promise, especially the combination strategies |
| AS, Angiosarcoma; ORR, Objective response rate; mPFS, median progression free survival; mOS, median overall survival; PR, Partial response; SD, Stable disease; PD, Progressive disease; TKIs, Tyrosine kinase inhibitors | | | | | |
