# Supplementary material for: Clinicopathological features, treatment outcomes, and prognostic factors of angiosarcoma: a 21-year experience at one center
Source: Orphanet J Rare Dis. 2025 Jun 11;20:298. doi: 10.1186/s13023-025-03819-9 (PMC12153173; doi:10.1186/s13023-025-03819-9)
Supplement: Supplementary file 4 — Supplementary Material 4. [file 13023_2025_3819_MOESM4_ESM.docx]

| **Table S4 Summary of key findings on immunotherapy for metastatic AS** | | | | | |
| --- | --- | --- | --- | --- | --- |
| **Study/Year** | **Patients** | **Treatment** | **No.** | **Key Findings** | **Notes** |
| ANGIOCHECK ^(56)^, 2024 | Cutaneous, Taxane-resistant AS | Nivolumab | 23 | ORR 28.6% (TMB-low) vs. 14.3% (TMB-high) | TMB may not predict response |
| NCT02834013 ^(57)^, 2021 | Metastatic/unresectable AS | Ipilimumab + Nivolumab | 16 | ORR 25% (60% in cutaneous); 1/7 TMB-high (PR); 2/3 PD-L1-high expression (1 PR) | Promising for cutaneous AS |
| NCT02815995 ^(58)^, 2022 | Advanced/metastatic AS | Durvalumab + Tremelimumab | 5 | ORR: 20% | Small sample size |
| NCT03512834 ^(59)^, 2024 | Unresectable AS | Paclitaxel + Avelumab (first-line) | 32 | ORR: 50%; mPFS: 6 mo, mOS: 14.5 mo | Promising first-line option |
| A091902 ^(60)^, 2024 | Taxane-naïve AS | Paclitaxel + Nivolumab vs. Paclitaxel | 62 | ORR: 33% vs. 34%; mPFS: 7.2 vs. 8.3 mo; mOS: 18 vs. 23 mo (all); ORR: 73% vs. 38%; mPFS: 16 vs. 8.3 mo (scalp/face); ORR: 11% vs. 26%; mPFS: 5.5 vs. 6 mo (non-scalp/face) | Site-specific benefit in cutaneous AS |
| Current study | Metastatic/unresectable AS | chemotherapy (4 paclitaxel, 1 doxorubicin, 1 temozolomide) + PD-1 inhibitors (first-line) | 6 | 2 SD and 4 PD; 1/4 PD-L1-high expression (poor response to later-line immunotherapy) | Less efficacy compared to prior studies |
| AS, Angiosarcoma; ORR, Objective response rate; mPFS, median progression free survival; mOS, median overall survival; SD, Stable disease; PD, Progressive disease | | | | | |
